# Hypomanic symptoms and first manic episode during ketogenic diet treatment in a patient taking venlafaxine: a case report with longitudinal glucose-ketone index and clinical monitoring

**DOI:** 10.3389/fnut.2026.1873930

**Published:** 2026-07-16

**Authors:** Marta Pou-Álvarez, Francesc Casanovas, David Córcoles, Jordi León-Caballero, Agnès Sabaté, Anna Maria González-Fresnedo, Pilar Samos, Víctor Perez-Sola, Luis Miguel Martín

**Affiliations:** 1 Department of Psychiatry, Complexo Hospitalario Universitario de Pontevedra e O Salnés, Pontevedra, Spain; 2 Mental Health Institute, Hospital del Mar, Barcelona, Spain; 3 Centro de Investigación Biomédica en Red, Área de Salud Mental (CIBERSAM), Madrid, Spain; 4 Department of Psychiatry and Forensic Medicine, Universitat Autònoma de Barcelona, Barcelona, Spain; 5 Hospital del Mar Medical Research Institute, Barcelona, Spain; 6 Department of Medicine and Life Sciences (MELIS), Pompeu Fabra University, Barcelona, Spain

**Keywords:** bipolar disorder, ketogenic diet, ketosis, metabolic psychiatry, mood disorders

## Abstract

**Background:**

The ketogenic diet (KD) is gaining increasing interest as a metabolic intervention for neuropsychiatric disorders, including mood disorders. However, its potential role in clinically relevant affective changes, such as hypomania or mania, remains insufficiently characterized.

**Methods:**

We present the case of a woman with a previous history of major depressive episode with psychotic symptoms and a posterior history of recurrent episodes of mild depression, who never achieved complete symptom remission. In an effort to ameliorate her chronic depressive symptoms, the patient started a KD for approximately 6 months. Metabolic monitoring included daily capillary measurements of glucose and ketone levels, with calculation of the glucose–ketone index (GKI) to assess ketosis and dietary adherence. Anthropometric parameters and physiological variables were recorded longitudinally. Laboratory assessments were performed before, during, and after the intervention. Psychiatric outcomes were evaluated using clinical scales, including the YMRS, PANSS, CGI, GAF, WHODAS and OAS, allowing assessment of symptom severity and clinical evolution. Pharmacologic, dietary, substance-related, sleep-related, and psychosocial exposures were reconstructed across clinically relevant phases.

**Results:**

After KD initiation, the patient reported improved mood and vitality, increased energy, reduced need for sleep, slight euphoria, and increased goal-directed activity. This period was retrospectively interpreted as possible hypomania. Four months after KD initiation, in the context of ongoing venlafaxine treatment (112.5 mg/day), absence of mood-stabilizing treatment, psychosocial stressors, reduced sleep and increased alcohol intake, and possible dietary drift, she developed a manic episode with psychotic features requiring urgent psychiatric care and home hospitalization. Symptoms remitted after antipsychotic treatment, lithium initiation, venlafaxine dose reduction, and temporary KD interruption. After remission, the patient chose to restart a less intensive KD under close supervision.

**Conclusion:**

This case describes hypomanic symptoms and subsequent mania temporally associated with KD in a patient taking venlafaxine with prior antidepressant-associated hypomanic symptoms and later diagnostic reclassification to bipolar I disorder. To our knowledge, it is the first report providing a longitudinal characterization of metabolic parameters in relation to the clinical course of mania. The case highlights the need to monitor mood, sleep, alcohol use, antidepressant exposure, dietary adherence, and ketosis when KD is considered in mood-disorder populations.

## 1 Introduction

Numerous pharmacological treatments are available for bipolar disorder. However, despite treatment, nearly half of all patients continue to experience recurrent mood episodes and complete remission is rare (1). The pharmacological treatment of this disorder has greatly improved in recent years, yet many patients continue to experience persistent symptoms and/or unwanted treatment-related side effects (2). In this context, the ketogenic diet (KD) has emerged as an alternative dietary approach to bipolar disorder. KD has shown potential benefits in several neuropsychiatric conditions, including bipolar disorder (3, 4).

The KD—a high fat, moderate protein, low carbohydrate diet (5)—was first introduced in 1921 as a treatment for refractory epilepsy. This diet has proven effective in several neurological and psychiatric conditions (6). The nutritional composition of the KD can induce a metabolic state known as ketosis in which the body uses ketones—instead of glucose—as an energy source (5). The KD has been shown to promote weight loss and improve metabolic health. It has also been shown to positively influence neurotransmission and neuroinflammation, two key processes implicated in the pathophysiology of bipolar disorder (7). The KD may also have antioxidant and anti-inflammatory effects by reducing the production of reactive oxygen species (7–9). The KD appears to regulate glutamatergic transmission and control of glutamatergic toxicity, thus improving neurotransmission (7).

Recent studies have extended the potential applications of the KD beyond epilepsy to other neurological and psychiatric disorders, including migraines, Parkinson’s disease, dementia, schizophrenia (10), brain tumours, and multiple sclerosis (11). The KD alters brain metabolism, leading to a switch from glucose as the primary fuel to fats (ketone bodies) produced by the liver, which have neuroprotective and anti-seizure properties (6, 11, 12). Several case series and observational studies have found that KD may promote symptom remission and mood stabilization in patients with bipolar disorder (1, 13). The findings of those studies have prompted interest in performing clinical trials to obtain more robust evidence (14).

A recent publication has reported the emergence of hypomanic and manic symptoms following KD initiation, including in individuals without prior bipolar-spectrum disorders (15, 16). However, evidence on the risks and benefits of KD in bipolar disorder remains limited. We report a case of a woman with recurrent depressive episodes who developed hypomanic symptoms after initiating a prescribed KD and a subsequent manic episode while taking venlafaxine. This case provides a detailed longitudinal characterization of glucose levels, ketone bodies, and the glucose-ketone index (GKI), together with a structured reconstruction of medication, dietary, substance, sleep, and psychosocial exposures across the clinical course.

## 2 Case presentation

### 2.1 Clinical background

V. is a 46-year-old Caucasian woman who lives in Barcelona. She is single, with a university level of education, and currently works in a position of high responsibility. She is actively involved in volunteer activities in Barcelona and regularly practices sports. She has no history of substance abuse and only occasionally uses alcohol in social contexts. She has no significant medical or surgical history, except for androgenetic alopecia. She has no family psychiatric history.

The patient reports having suffered anxiety attacks in her early youth. In 2013, she presented her first major depressive episode (MDE), which was accompanied by psychotic symptoms. She was successfully treated with venlafaxine (150 mg/day). She was also prescribed olanzapine but did not take it. During recovery, she presented mild hypomanic symptoms that resolved quickly without the need to reduce the antidepressant dosage. She continued psychiatric follow-up at a private clinic, maintaining venlafaxine at doses ranging from 37.5 to 150 mg/day. Attempts to decrease or discontinue the antidepressant coincided with mild depressive relapses characterized by low energy, fatigue, and a slight loss of volitional capacity that did not interfere with her work activity. Complaints of low energy and low mood persisted even after reintroduction of higher venlafaxine doses (up to 150 mg/day). She did not receive mood stabilizers or other pharmacological treatments during this period.

Outside this brief period of antidepressant-associated mild hypomanic symptoms during recovery from the 2013 depressive episode, no other hypomanic or manic episodes were documented during the following years. However, a latent bipolar-spectrum disorder was considered but never formally confirmed during this period.

### 2.2 Ketogenic metabolic therapy intervention strategy

In July 2023, she began follow-up care at an integrative medical center where she sought help for her mild but chronic depressive symptoms. At this center, she was under the care of a psychiatrist and a nutritionist. Based on the advice of the medical team, she began a professionally supervised KD pattern (high fat, moderate protein, low carbohydrate diet). This protocol specifically prohibited processed foods, sugar, cereals and their derivatives, legumes, and potatoes. Instead, she was instructed to cook foods with butter or lard and to prioritize foods such as eggs, meats, natural oils (e.g., coconut or medium-chain triglyceride [MCT] oils), nuts, and fats such as those from avocado. Specifically, her baseline weekly dietary plan included approximately 50 g/day of supplemental fats (25 g/day of butter and 25 g/day of MCT oils), together with eggs, extra-virgin olive oil, avocado, nuts (including Brazil nuts and macadamia nuts), Parmesan cheese, cured and cooked meats, seafood (primarily squid and octopus), and small portions of low-carbohydrate vegetables such as cucumber, spinach, and zucchini, while maintaining a very low carbohydrate intake. The available records did not include a fixed ketogenic ratio, a precise carbohydrate target in grams/day, or a formal calorie prescription; this is reported as a limitation of the intervention description. The dietary intervention, concurrent psychotropic exposure, alcohol use, and clinical phases are summarized in Table 1.

**Table 1:** Structured timeline of dietary, pharmacologic, substance, sleep-related, and clinical exposures.

Clinical phase	Diet/metabolic status	Medication	Food/ Substances/ supplements/other diet adjuncts	Clinical course and causal interpretation
2013 MDE with psychotic symptoms and recovery	No KD	Venlafaxine 150 mg/day. Olanzapine prescribed but not taken. No mood stabilizer. Other medication: Not documented	Substance use: very occasional alcohol use Caffeine/stimulants: not documented Supplements: none reported	MDE remitted. Mild hypomanic symptoms occurred during recovery while treated with venlafaxine and resolved without dose reduction; relevant competing explanation for later mood activation.
2013–July 2023	No KD	Venlafaxine maintained at 37.5–150 mg/day; no mood stabilizer. Other medication: Not documented	Substance use: very occasional alcohol use Caffeine/stimulants: not documented Supplements: none reported	Recurrent mild depressive relapses when venlafaxine was reduced or discontinued; persistent low energy and low mood despite higher venlafaxine doses.
July–December 2023: KD initiation	KD pattern: high-fat, moderate-protein, very-low-carbohydrate diet During July–October 2023, low GKI (<3) supports ketosis.	Venlafaxine 112.5 mg/day unchanged; no mood stabilizer. Other medication: none reported.	Food excluded: processed foods, sugars, cereals, legumes, and potatoes. Food Included: 25 g/day butter, 25 g/day MCT oil, eggs, olive oil, avocado, nuts, cheese, cured/cooked meats, seafood and small portions of low-carbohydrate vegetables. Caffeine/stimulants: none reported. Substance use: very occasional alcohol use Supplements: Omega 3 + 6 (dose not addressed) MCT oil 25 g/day	Improved mood and vitality, increased energy, reduced need for sleep, slight euphoria, new projects, faster speech, intermittent irritability. Retrospectively compatible with hypomania, but antidepressant activation/recovery/prodrome also plausible.
December 2023–January 2024: prodromal worsening	GKI rose, indicating loss of ketosis.	Venlafaxine 112.5 mg/day unchanged; no mood stabilizer. Other medication: none reported.	Substance use: Alcohol intake increased (2–3 standard drinks/day) during the week before emergency presentation. Supplements: Omega 3 + 6 (dose not addressed)	Work/personal stressors, sleep reduced to approximately 3 h/day, clinical activation progressed. Rising GKI may be cause, consequence, or marker of prodrome.
Emergency care and HHT, February–March 2024	KD intentionally interrupted; high GKI/low ketosis during treatment period.	Olanzapine 10 mg acutely, then 20 mg/day; switch to aripiprazole 10 mg/day; lithium carbonate 1,000 mg/day; venlafaxine reduced to 75 mg/day. Other medication: none reported.	Caffeine/stimulants: none reported. Substance use: Alcohol avoidance. Supplements: Omega 3 + 6 (dose not addressed)	Manic episode with psychotic features remitted. Metabolic values in this phase are confounded by medication changes and intentional KD interruption.
After HHT discharge, April 2024 onward	Less intensive KD restarted under nutritional supervision; lower GKI values in April 2024.	Venlafaxine 75 mg/day Aripiprazole 10 mg/day Olanzapine 5 mg/day Lithium 1,000 mg/day Other medication: none reported.	Caffeine/stimulants: none reported. Substance use: Alcohol avoidance Supplements: Omega 3 + 6 (dose not addressed)	Patient perceived benefits in vitality and mood and chose to continue KD despite the preceding admission, within a shared decision-making framework.

HHT, Home Hospitalization Team; KD, ketogenic diet; GKI, glucose-ketone index; MDE, major depressive episode; MCT, medium-chain triglyceride.

**Figure 1:**
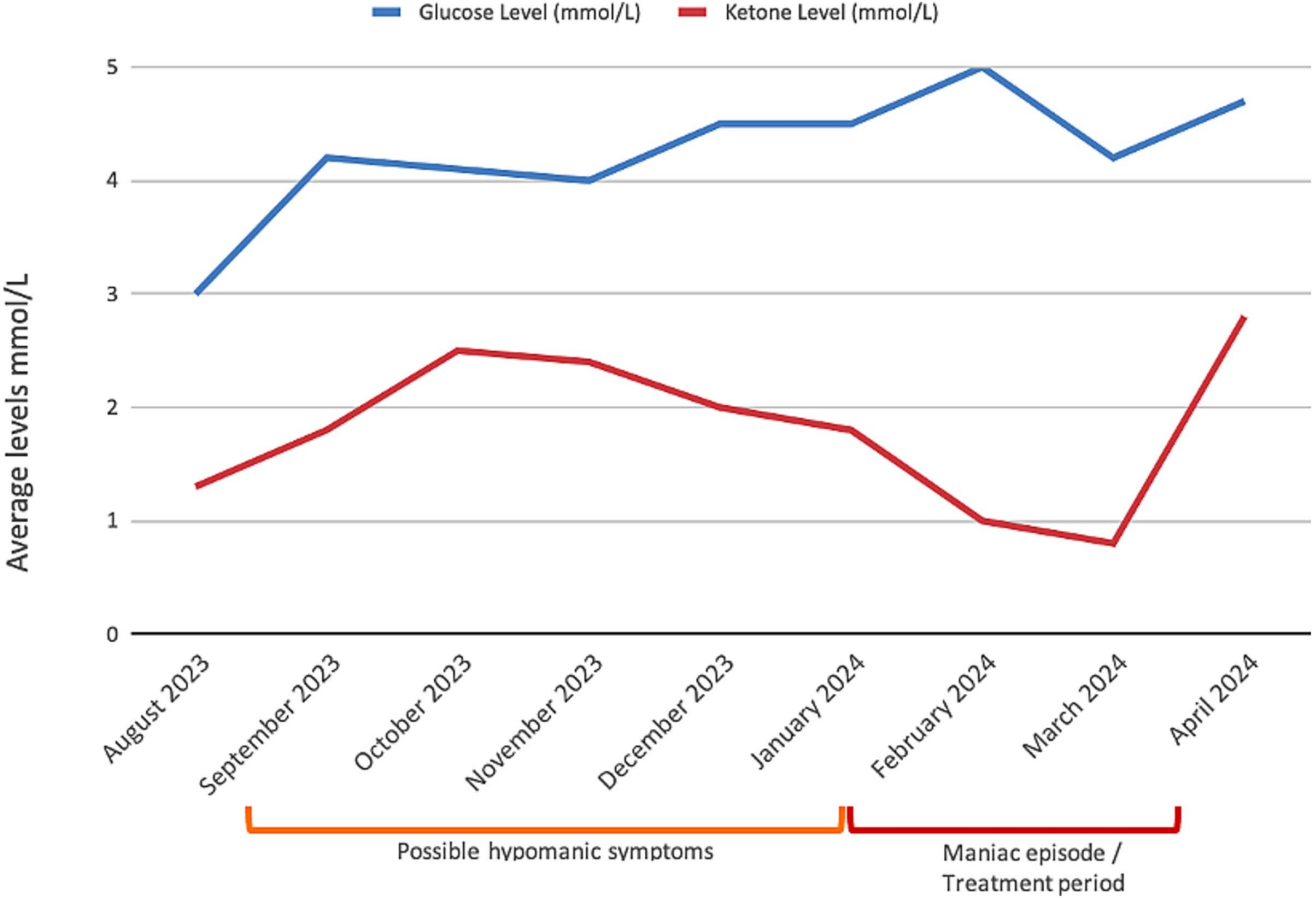
Longitudinal changes in capillary blood glucose and ketone levels across the clinical phases.

**Figure 2:**
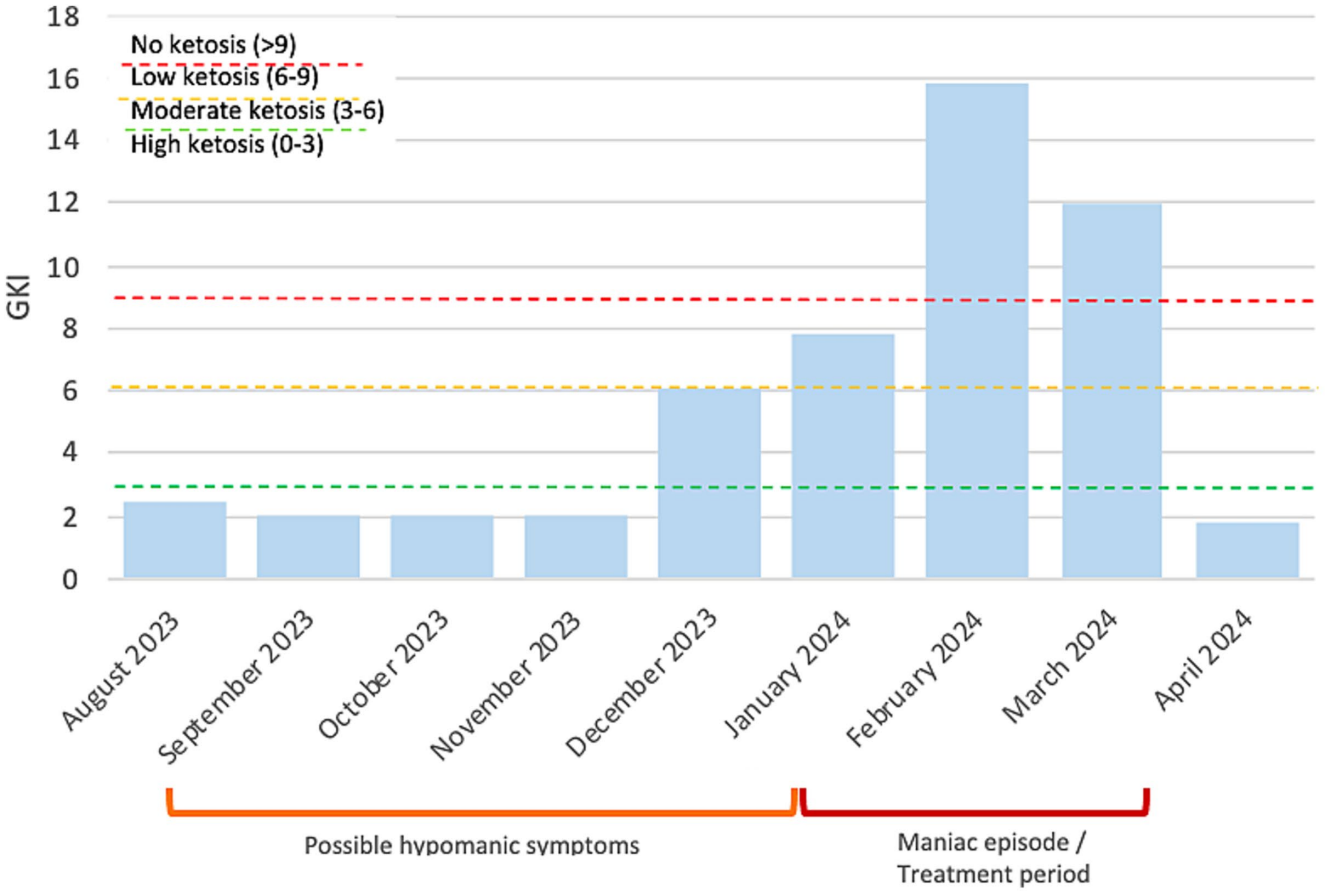
Longitudinal changes in the glucose–ketone index (GKI) across the clinical phases.

Metabolic monitoring included blood tests, blood pressure measurements, and body weight measurements (Table 2). Glucose and ketones were measured daily with a capillary blood meter (Keto-Mojo; Napa, CA, United States) (Figure 1). This device measures *β*-hydroxybutyrate, a ketone body. These data were then used to calculate the glucose-ketone index (GKI), a metric that measures the level of ketosis (Figure 2). No sudden changes in weight or significant weight loss were observed during the 6-month period she was on the KD. Her weight remained relatively stable (± 1 to 2 kg) over this time period, which has been more frequently described among women following a KD (17). The patient stated that she strictly followed this diet during the first few months, but especially during the period from July to October 2023. The low mean GKI levels (< 3) recorded during this period seems to confirm this.

**Table 2:** Anthropometric measurements and blood test analyses before initiating the ketogenic diet, during the diet, and after temporary discontinuation.

Parameters	Unit of measurement / reference range	Pre-KD	During KD	Post-KD
Anthropometric measurements				
Weight	kg	56	55 ↓	54 ↓
Height	cm	162.3	162.3	162.3
BMI	index (healthy range 18.5–24.9)	21.26	20.9 ↓	20.5 ↓
Systolic blood pressure	mmHg	121	Not available	Not available
Diastolic blood pressure	mmHg	81	Not available	Not available
Heart rate	beats/min	83	Not available	Not available
Haematology				
Red blood cells	10E6/L	3.77	4.05	3.84
Haemoglobin	g/dL	12.2	13.3	12.8
Haematocrit	%	36.4	39.5	36.1
MCV	fL	96.8	97.5	94.0
MCH	pg	34	32.8	33.3
CHCM	g/dL	36	33.7	35.5
MPV	fL	10.3	10.7	11.0
Leukocytes	10E3/L	5.88	4.23	4.0
Neutrophils	%	Not available	50.9	49.4
Neutrophils	10E3/L	Not available	2.15	1.98
Lymphocytes	%	Not available	38.8	41.0
Lymphocytes	10E3/L	1.87	1.64	1.64
Monocytes	%	Not available	9.2	8.3
Monocytes	10E3/L	0.39	0.39	0.33
Eosinophils	%	0.9	0.9	0.8
Eosinophils	10E3/L	0.1	0.04	0.03
Basophils	%	0.5	0.2	0.5
Basophils	10E3/L	0.02	0.01	0.02
Platelets	10E3/L	275	286.0	270.0
Prothrombin time	s	Not available	Not available	142.0
INR	Not available	Not available	Not available	0.83
Partial thromboplastin time	s	Not available	Not available	0.99
Biochemistry				
Glucose	mg/dL (fasting reference 70–99)	77	86	100 ↑
Ferritin	ng/mL	Not available	53	81
Creatine kinase	U/L	Not available	Not available	105
Aspartate aminotransferase	U/L	31	37	33
Alanine aminotransferase	U/L	32	37	33
Gamma-glutamyl transferase	U/L	17	13	14
Alkaline phosphatase	U/L	Not available	48	48
Total bilirubin	mg/dL	Not available	0.37	0.18
Lipase	U/L	Not available	Not available	65.4
Alpha-amylase	U/L	Not available	Not available	108
Urea	mg/dL	22	22.5	32.2
Creatinine	mg/dL	0.62	0.64	0.71
Urate	mg/dL	2.7	2.46	2.73
Total proteins	g/dL	Not available	Not available	7.0
Calcium	mg/dL	Not available	Not available	9.6
Sodium	mmol/L	Not available	140	142
Potassium	mmol/L	Not available	4.16	4.02
Chloride	mmol/L	Not available	100	104
Iron	mcg/dL	Not available	Not available	84.9
Ferritin	ng/mL	Not available	Not available	53
Transferrin	mg/dL	Not available	Not available	300
Cholesterol	mg/dL (desirable <200)	175	298 ↑	240 ↑
HDL cholesterol	mg/dL (women: >50; ≥60 favorable)	55	116 ↑	101 ↑
LDL cholesterol	mg/dL (optimal <100; high ≥160)	104	187 ↑	146 ↑
Triglycerides	mg/dL (normal <150)	76	69 ↓	42 ↓
Zinc	mcg/dL	Not available	Not available	99.9
Copper	mcg/dL	Not available	Not available	90.8
Magnesium	mg/dL	Not available	Not available	2.03
Folic acid	ng/mL	Not available	8.1	7.3
Hormones				
Thyrotropin	mcIU/mL	Not available	Not available	3.05
Vitamin B12	pg/mL	Not available	520	508
Vitamin D (25-OH)	ng/mL	Not available	Not available	63

KD, ketogenic diet; BMI, body mass index; MCV, mean corpuscular volume; MCH, mean corpuscular haemoglobin; CHCM, cellular haemoglobin concentration mean; MPV, mean platelet volume; INR, international normalized ratio; HDL, high density lipoprotein; LDL, low density lipoprotein. “Not available” indicates that the parameter was not documented for that phase. Reference ranges are shown for the most clinically relevant cardiometabolic variables. Values with ↑ indicate values above the reference range or a clinically relevant increase; ↓ indicates a clinically relevant decrease from baseline. HDL increases and triglyceride/BMI decreases are shown directionally because they may be metabolically favorable despite concurrent LDL-C and total-cholesterol increases.

### 2.3 Evaluation of intervention

Shortly after starting the diet, the patient experienced a subjective improvement in mood. This period was retrospectively interpreted as possible hypomania. She described increased energy, a reduced need for sleep, slight euphoria, and changes in habits, including the initiation of new projects. Her friends also identified a change in her behavior, reporting that she started speaking faster than usual and was more easily irritated by others at times. However, no YMRS or structured mania assessment was performed prospectively during the initial four-month period. Therefore, alternative interpretations must be considered, including antidepressant-associated activation, recovery from chronic depressive symptoms, increased productivity related to improved energy, and an early bipolar prodrome. During this entire period, she maintained her regular dose of venlafaxine (112.5 mg/day) with no modifications and was not receiving a mood stabilizer (Table 1).

The patient maintained work and social functioning during this period, which is consistent with hypomania but also reinforces the need for diagnostic caution. In January 2024, she reported several new work and personal life-related stressors. One week before seeking medical help, she started sleeping less (only three hours per day) and began drinking alcohol more frequently. In the 48 h before presenting to the psychiatric emergency department, she developed several psychotic symptoms: her speech became disorganized and she began making loose associations about her past mixed with interpretative and referential delusional content (she believed she was involved in a game in which she had to overcome various challenges). Her friends accompanied her to the emergency department, where she was admitted and immediately given 10 mg of olanzapine.

The acute episode met criteria for a manic episode with psychotic features, based on elevated mood, decreased need for sleep, increased goal-directed activity, disorganized speech, irritability, and psychotic symptoms. Given the history of recurrent depression and the current manic episode, the longitudinal diagnosis was revised to bipolar I disorder. Clinical assessment was supported by structured rating scales during admission and follow-up (Table 3), including the Young Mania Rating Scale (YMRS), Positive and Negative Syndrome Scale (PANSS), Clinical Global Impression (CGI), Global Assessment of Functioning (GAF), World Health Organization Disability Assessment Schedule (WHODAS 2.0), and Overt Aggression Scale, which showed significant improvement after treatment.

**Table 3:** Clinical scales at admission and discharge from the Home Hospitalization Team.

Scale	At admission	At discharge
Overt Aggression Scale	5	Not assessed
World Health Organization Disability Schedule 2.0	18	Not assessed
Clinical Global Impression - Severity of illness	4 (Moderately ill)	Not assessed
Clinical Global Impression - Global improvement	Not applicable	1 (Very much better)
Global Assessment of Functioning	65	95
PANSS Total score	45	34
PANSS Positive Scale	16	7
PANSS Negative Scale	9	8
PANSS General Psychopathology Scale	20	19
Young Mania Rating Scale	13	0

PANSS, Positive and Negative Syndrome Scale.

### 2.4 Follow-up and outcomes

Following the admission, the patient exhibited partial insight into her delusional symptoms, although the other manic symptoms were still present. Given her solid support network of friends, she was referred to the Home Hospitalization Team (HHT) at the Hospital del Mar (Barcelona). The HHT is a psychiatric outpatient program in which a home care team (a psychiatrist and mental health nurse) perform at-home follow-up visits of patients experiencing an acute crisis of mental disorder. This program is described in detail elsewhere (18).

During the first few weeks of follow-up, the patient received olanzapine 20 mg/day. Later, she was switched to aripiprazole 10 mg/day. Gradually, she exhibited complete insight into the delusional symptoms. Affective symptoms and sleep architecture improved over time. She received psychoeducation regarding manic symptoms, relapse prevention, sleep regularity, alcohol avoidance, and the need for close monitoring if dietary ketosis was restarted. Her previous psychiatric history was reassessed, leading to reclassification of the acute episode as a manic episode with psychotic symptoms and to a longitudinal diagnosis of bipolar I disorder. Consequently, lithium carbonate was prescribed (1,000 mg/day), with a plasma level of 0.75 mEq/L at discharge.

The venlafaxine dose was lowered to 75 mg/day, but the patient was reluctant to stop it completely because previous dose reductions had been followed by depressive relapses. She agreed to start lithium and to attempt venlafaxine withdrawal in the following months. Before discharge from the HHT, olanzapine was reduced to 5 mg/day with the intention of discontinuing it at the next follow-up visit, while aripiprazole was maintained at 10 mg/day.

At discharge from the HHT intervention, the patient was euthymic and showed progressive global recovery of her usual work and social activities. The scores on the clinical scales at admission and discharge from the Home Hospitalization Team are shown in Table 3.

The patient agreed to interrupt the KD during the home hospitalization period (February and March 2024), given the uncertainty regarding its potential in the onset of the manic episode. The period of low ketosis coincided with venlafaxine dose reduction, as well as the initiation of lithium and antipsychotic treatment. Therefore, multiple factors co-occurred during the patient’s clinical recovery. At discharge from the HHT, she was taking a mood stabilizer with a reduced venlafaxine dose. After discussing the potential benefits and risks of resuming the ketogenic diet, the patient decided to restart a less intensive version of the diet based on her previous dietary plan, under close psychiatric and nutritional supervision, as evidenced by lower GKI values in April 2024 (Figure 2).

### 2.5 Patient perspective

*“The KD was recommended to me by an integrative psychiatrist whom I had consulted to improve my energy, and indeed the most significant change I noticed was a major increase in my energy levels, especially in the mornings. It was not very different from the effect venlafaxine had on me during the first six months I took it. The manic and psychotic symptoms appeared suddenly, although in the preceding period people around me had noticed that I seemed somewhat euphoric. During the home hospitalization, I did not continue the diet after being admitted to the emergency room because I was afraid, but in my opinion, it can be a valid tool if carried out under constant supervision.”*

## 3 Discussion and conclusions

This case describes hypomanic symptoms and a subsequent first manic episode with psychotic features occurring during KD treatment in a patient taking venlafaxine, with prior mild antidepressant-associated hypomanic symptoms. Importantly, the manic episode occurred during a period characterized by psychosocial stress, reduced sleep, increased alcohol intake, and possible dietary drift or loss of ketosis. Therefore, the case should be interpreted as a temporally associated, multifactorial mood-switch episode, in which the specific contribution of the KD remains uncertain. Palmer recently reported a case series of individuals who developed hypomania or mania shortly after initiating a KD, most of whom had no prior bipolar-spectrum diagnosis (15). The novelty of the present report is not the mere occurrence of hypomanic/manic symptoms occurring during KD treatment in pharmacologically complex patients, but the longitudinal single-case characterization of glucose, ketone bodies, and GKI in relation to the clinical course and treatment phases.

The patient had a previous diagnosis of recurrent depressive disorder and had not experienced a full manic episode before, but she had experienced mild hypomanic symptoms during recovery from depression while receiving venlafaxine. Venlafaxine is a clinically plausible contributor to the described mood switching. The patient was taking 112.5 mg/day throughout the period in which symptoms emerged, had a prior history of mild hypomanic symptoms during recovery from depression while receiving venlafaxine, and was not receiving a mood stabilizer. Although the patient remained on a stable antidepressant dose, this does not exclude the possibility of interaction, potentiation, or altered vulnerability following a major metabolic intervention. In bipolar depression, venlafaxine has been associated with a higher risk of switching into hypomania or mania than bupropion or sertraline in comparative studies (19). A recent systematic review—comprised primarily of preclinical and observational studies—assessed the potential benefits of KD for mood and anxiety disorders, finding promising antidepressant and mood-stabilizing effects (20).

KD has shown potential symptom benefits in psychiatric disorders in case reports, observational studies, and early trials (3, 16). For example, numerous studies have found that KD reduces hallucinations in psychotic disorders (3, 13, 21–24). In recent years, interest in evaluating the potential role of KD in bipolar disorder has continued to grow, although the evidence base remains limited. Previous case reports and online survey data have suggested that KD may improve energy, concentration, and clarity of thought, promote weight loss, and/or reduce the frequency of episodes in rapid cycling disorders (13, 25). A controlled study involving members of online bipolar disorder forums found that 56.4% (93/165) of individuals who followed KD reported mood stabilization or remission of symptoms (25). A semi-controlled retrospective analysis of 12 patients with bipolar disorder found a significant reduction in depressive symptoms (1). A pilot trial involving patients with bipolar disorder or schizophrenia who followed KD for four months showed reduced illness severity, increased life satisfaction, better global functioning, and better sleep quality (3). These findings justify further study but should not be interpreted as establishing efficacy or safety profiles for all patients with mood disorders.

In line with recent reports, objective metabolic monitoring appears to be a relevant methodological component. Adherence to ketogenic interventions has been documented using glucose and ketone blood markers, reinforcing the importance of metabolic tracking in psychiatric applications (10). Structured clinical scales have also been used to describe symptom changes before and after KD initiation, in some cases retrospectively (26). Ketone body measurements have been proposed as useful tools both to verify adherence and to individualize dietary interventions (27).

The antidepressant and mood stabilizing effects of KD may be due to modulation of ion channels, an increase in GABA concentration, and the positive influence on glutamate metabolism and nerve cells (28). Studies conducted in young adults and adolescents with affective disorders and epilepsy have shown that the KD modifies dopamine and serotonin levels (29). From a biomechanical perspective, KD may stabilize mood by reducing oxidative stress, improving mitochondrial biogenesis, and reducing inflammation (1, 3, 30). The KD has also been shown to increase neurotrophic factors such as brain-derived neurotrophic factor (31) and to stabilize the neural network (5). Other systemic effects of KD, including better insulin sensitivity and reduced inflammation, could also benefit the course of affective disorders (21, 31). A recent review discussed how KD may work in patients with mania/hypomania through interactions with mitochondrial function and dopaminergic and GABAergic neurotransmission abnormalities (32).

In the present case, the presence of multiple co-occurring factors does not allow establishing a definitive causal hierarchy, including the specific contribution of the KD. The relevant exposure change was KD initiation added to ongoing venlafaxine in a patient with prior antidepressant-associated hypomanic symptoms and without mood-stabilizing treatment. We therefore consider several competing and potentially interacting factors: KD-related activation, antidepressant-associated activation, KD-antidepressant interaction, latent bipolar disorder, sleep reduction, psychosocial stress, increased alcohol use, and dietary drift/loss of ketosis. To our knowledge, only a limited number of reports have described similar affective switches in this context, particularly in patients without a previous bipolar-spectrum diagnosis (1, 3, 15, 25).

Smolensky et al. reported that KD may be more efficacious in premenopausal women, raising the possibility that sex- and reproductive-stage-related factors could influence response to ketogenic metabolic interventions (5). However, this remains speculative and cannot be inferred from a single case.

Interestingly, in the month preceding the manic episode, the patient’s GKI increased substantially, suggesting reduced adherence to the ketogenic intervention or loss of sustained ketosis. This temporal pattern complicates a simple dose–response interpretation in which deeper ketosis directly precipitated the manic episode. One possible, although speculative, interpretation is that the clinical effects of KD may have differed across phases: initial ketosis may have coincided with increased energy, reduced need for sleep, and potential hypomanic symptoms, whereas later dietary relaxation or loss of ketosis may have contributed to further mood destabilization in a vulnerable patient. However, this interpretation remains uncertain because several concurrent factors were present, including ongoing venlafaxine treatment, previous antidepressant-associated hypomanic symptoms, psychosocial stress, sleep reduction, increased alcohol use, and absence of mood-stabilizing treatment. Therefore, GKI levels cannot be interpreted solely as reflecting the physiological effects of the KD.

The metabolic data can therefore be interpreted in more than one direction. First, sustained ketosis during the initial months may have contributed to increased energy, reduced need for sleep, and affective activation. Second, rising GKI values before the acute manic episode may suggest poorer adherence or loss of ketosis, raising the possibility that dietary drift or discontinuation of a previously rigorous KD contributed to destabilization. Third, emerging activation, stress, sleep reduction, and increased alcohol use may themselves have worsened dietary adherence, making rising GKI an effect or marker of prodrome rather than a cause. Fourth, GKI values during February and March 2024 are confounded by acute psychiatric treatment, intentional KD interruption, antipsychotic exposure, lithium initiation, and venlafaxine dose reduction. These competing interpretations prevent any simple conclusion that either high ketosis or KD discontinuation was the primary cause of mania.

This complex situation is consistent with emerging reports suggesting that both KD initiation and KD discontinuation may coincide with clinically relevant changes in activation, sleep, and mood in susceptible individuals. In a pilot study of KD in euthymic patients with bipolar disorder, one participant developed hypomania lasting 30 days, requiring a reduction in fluoxetine dose (33). Dietch et al. (20) reported psychiatric decompensation after abrupt discontinuation of a previously rigorous KD, and another report described hypomanic symptoms emerging within one week of KD discontinuation and resolving spontaneously after 3–4 weeks (24). In a previous bipolar I case report, KD was associated with reduced anxiety and sustained euthymia, although the patient described brief, self-limited episodes of activation and irritability rather than syndromal hypomania (22). Worsening of positive and negative symptoms after KD discontinuation has also been described in patients with schizoaffective disorder (23). Taken together, these observations suggest that changes in ketogenic adherence may be clinically relevant, but they do not establish that either KD initiation or discontinuation independently causes manic relapse. The role of concomitant antidepressants, sleep disruption, alcohol use, psychosocial stressors, and underlying bipolar vulnerability must therefore be carefully considered.

Beyond psychiatric outcomes, the present case also raises relevant cardiometabolic monitoring issues. In this patient, total cholesterol and low-density-lipoprotein (LDL) cholesterol increased during KD and remained above baseline after temporary discontinuation, which may represent a treatment-related cardiovascular safety signal. At the same time, high-density-lipoprotein (HDL) cholesterol increased, triglycerides decreased, and body mass index decreased, changes usually considered metabolically favorable. Current literature suggests that KD may improve body weight, triglycerides, glycemic indices, and sometimes HDL cholesterol, but may also increase LDL cholesterol and total cholesterol in some individuals (34–36). The long-term cardiovascular implications remain uncertain and are likely influenced by baseline risk, genetics, saturated fat intake, fiber intake, and whether apoB/non-HDL cholesterol also increase (34–36). An observational UK Biobank analysis of low-carbohydrate high-fat dietary patterns found higher LDL-C and apoB levels and higher incident major adverse cardiovascular events, although such data cannot prove causality and the dietary pattern was not identical to a clinically supervised KD (35). In the present case, the cardiometabolic changes were discussed with the patient, leading to a plan for serial lipid monitoring and dietary modification toward a more cardioprotective fat profile rather than unrestricted saturated-fat intake.

Sleep disruption may be another clinically relevant pathway. Palmer’s 2026 case series described decreased need for sleep as a prominent symptom after KD initiation; several participants modified the diet or used sleep medication to restore sleep, and most continued KD because they perceived weight or mental-health benefits (15). A systematic review and meta-analysis of Ramadan diurnal fasting found a reduction in total sleep duration during fasting (37). Because KD can function as a fasting-mimicking metabolic intervention and sleep deprivation is a recognized precipitant of hypomania and mania in susceptible individuals (38), sleep reduction should be monitored explicitly when KD or fasting-like interventions are used in patients with mood-disorder vulnerability. In the present case, reduced sleep occurred both during the initial activation period and immediately before emergency presentation.

This sleep-mediated hypothesis has practical implications for treatment. In patients with known or possible bipolar vulnerability, KD initiation should include a baseline sleep assessment, prospective sleep diary or actigraphy when feasible, and predefined safety thresholds for sleep reduction. Clinically, this may require avoiding fasting windows or excessive caloric restriction, reducing the intensity of ketosis, adjusting the evening meal plan, pausing KD during emerging activation, prioritizing rapid sleep restoration, reconsidering antidepressant exposure, and ensuring mood-stabilizing treatment before rechallenge.

After discussing the potential risks and benefits of KD with the patient, we jointly decided to restart a less intensive KD while maintaining mood-stabilizing treatment and close psychiatric and nutritional monitoring. The plan included monitoring for early warning signs of affective activation, particularly reduced sleep, increased goal-directed activity, irritability, alcohol use, and changes in dietary adherence. Given the limited evidence in cases such as this one, continued follow-up may provide useful naturalistic information, but any future observations will remain confounded by concurrent pharmacological treatment and clinical monitoring.

Current expert recommendations emphasize the importance of careful monitoring when implementing ketogenic interventions in patients with severe mental illness. These include baseline metabolic assessment, regular laboratory follow-up, frequent ketone monitoring during initiation, and close psychiatric supervision, particularly in the early phases of treatment (16). In patients with depressive histories or possible bipolar vulnerability, monitoring should also include antidepressant exposure, sleep duration, alcohol use, emerging irritability/euphoria, and early changes in goal-directed activity. In patients who develop LDL-C or total-cholesterol elevations, individualized cardiovascular risk assessment, non-HDL cholesterol or apoB measurement when available, and diet-composition review should also be considered. These considerations support the clinical approach adopted in the present case, including close follow-up and the introduction of a mood stabilizer before reinitiating the diet.

The main limitation of this study is its single-case design, which lacks a control condition, limits generalizability, is susceptible to publication and selection bias, and does not allow temporal associations to be distinguished from causal relationships. The KD prescription did not include a fixed ketogenic ratio, a specific daily carbohydrate target, or a formal caloric prescription, limiting the precision with which the dietary intervention could be characterized. The initial hypomanic period was reconstructed retrospectively and was not assessed prospectively with YMRS or a structured diagnostic interview. Sleep duration, alcohol intake, dietary adherence, and several exposure variables were also reconstructed retrospectively and may be affected by recall bias. In addition, the manic episode occurred in a context of ongoing venlafaxine treatment, previous antidepressant-associated hypomanic symptoms, absence of mood-stabilizing therapy, psychosocial stress, reduced sleep, increased alcohol use, and changing GKI values. The timing of laboratory monitoring was not uniform across phases, and lipid changes cannot be attributed solely to KD without considering weight change, dietary composition, medication changes, and individual metabolic susceptibility. By contrast, strengths of the report include medical and nutritional supervision, daily capillary glucose and ketones monitoring, longitudinal GKI data, clinical rating scales during acute treatment, and explicit reconstruction of competing exposures.

The present study describes hypomanic symptoms and subsequent mania temporally associated with KD initiation in a patient receiving venlafaxine, with prior antidepressant-associated hypomanic symptoms and later diagnostic reclassification to bipolar I disorder. This case highlights an important research priority for the emerging field of metabolic psychiatry. It raises the possibility that ketogenic interventions may interact with concurrent psychotropic medication exposure, particularly antidepressants, in vulnerable patients. This case contributes to emerging evidence that clinically significant affective changes may coincide with KD initiation, fasting-mimicking metabolic states, or changes in dietary adherence in susceptible individuals, particularly in the context of antidepressant exposure, sleep disruption, alcohol use, psychosocial stress, and bipolar vulnerability. However, it does not establish that the KD was an independent causal factor in the onset of the manic episode. Future prospective studies of ketogenic interventions in psychiatric populations should systematically document concurrent psychotropic medication use, including antidepressant class, dose, and timing, as well as mood stabilizer status, sleep changes, alcohol consumption, and other relevant clinical exposures. Larger prospective studies are needed to clarify efficacy, incidence of adverse effects, and optimal monitoring strategies for patients with mood disorders initiating or discontinuing a KD.

## Data Availability

The datasets presented in this article are not readily available because of ethical and privacy restrictions. Requests to access the datasets should be directed to the corresponding author.
